# Rising Public Interest in Weight Loss Medications and Growing Awareness of Their Aesthetic Sequelae: An Infodemiologic Google Trends Analysis and Clinical Diagnostic Patterning

**DOI:** 10.1111/jocd.70670

**Published:** 2026-01-12

**Authors:** Alec D. McCarthy, Kay Durairaj, Jacob Linneman‐Heath, Dusan Sajic, Mara Dacso, Alan Durkin

**Affiliations:** ^1^ Global Medical Affairs Merz Aesthetics Raleigh North Carolina USA; ^2^ K. Kay Durairaj, M.D., A Medical Corp Pasadena California USA; ^3^ Derma Skin Institute Guelph Ontario Canada; ^4^ McMaster University Hamilton Ontario Canada; ^5^ Innovative Dermatology Plano Texas USA; ^6^ Ocean Drive Plastic Surgery, Dermatology, and MedSpa Vero Beach Florida USA; ^7^ Florida State University College of Medicine Tallahassee Florida USA

**Keywords:** biostimulators, glucagon‐like peptide‐1 receptor agonists, Google trends, Ozempic face, public interest

## Abstract

**Background:**

Glucagon‐like peptide‐1 receptor agonists (GLP‐1 RAs) have gained rapid popularity for both medical and consumer‐directed weight loss. This growth has been accompanied by increased public discussion regarding facial aesthetic changes, commonly referred to as “Ozempic face,” characterized by volume depletion and cutaneous laxity.

**Objective:**

To quantify temporal patterns of public search interest in a widely known GLP‐1 RA and evaluate corresponding awareness of its cosmetic facial sequelae using infodemiologic methods.

**Methods:**

Google Trends data for “Ozempic” and related frequently co‐searched queries were analyzed from November 2021 to December 2024. Trends in relative search volume (RSV) were examined, with particular focus on terms associated with facial aesthetics, including “Ozempic face” and “plastic surgeons Ozempic face.”

**Results:**

RSV for “Ozempic” showed a steady upward trajectory over the study period. Queries related to facial aesthetic consequences exhibited substantial proportional increases. Notably, “Ozempic face” demonstrated a 4600% rise in RSV, and searches for “plastic surgeons Ozempic face” similarly grew markedly.

**Conclusions:**

Public interest in GLP‐1 RAs is strongly associated with rising awareness and concern about their facial aesthetic effects. These trends suggest that aesthetic practitioners should expect more patient inquiries regarding GLP‐1–related facial changes and should proactively integrate counseling and corrective treatment options into clinical practice.

## 1 Introduction

Glucagon‐like peptide‐1 receptor agonists (GLP‐1RAs) have transformed medical and consumer approaches to obesity and weight loss. Following the June 2021 U.S. Food and Drug Administration (FDA) approval of the higher‐dose formulation for weight loss, utilization and off‐label prescribing surged [1, 2]. GLP‐1 RAs mimic endogenous glucagon‐like peptide‐1 (GLP‐1) to enhance glucose‐dependent insulin secretion, suppress glucagon release, slow gastric emptying, and promote satiety, thereby lowering blood glucose and facilitating weight loss [3].

Concurrently, beginning in early 2023, the lay term “Ozempic face” gained widespread traction. Mainstream media outlets, dermatologic commentaries, and patient‐oriented forums noted and pronounced facial volume depletion, cutaneous laxity, and a prematurely aged appearance, attributing these changes to the rapid adipose reduction achieved with GLP‐1RAs [4]. While the aesthetic community quickly proposed corrective interventions, from calcium hydroxylapatite (CaHA) and poly‐L‐lactic acid (PLLA) to surgical fat grafting and facelifting surgeries, objective data quantifying the extent of public concern and awareness of cosmetic sequelae have remained scarce [5].

Infodemiology, the study of online information patterns, offers a proxy for real‐time public sentiment [6]. Google Trends (GT; Google, Mountain View, CA, USA) aggregates anonymized search queries, providing relative search volume (RSV) indices that have been used to forecast disease outbreaks, track cosmetic‐procedure interest, and model on and off‐label drug‐utilization trends [7, 8, 9].

This study leverages GT to quantify temporal patterns of search interest in “Ozempic” (Ozempic, semaglutide; Novo Nordisk, Bagsvaerd, Denmark) and GT‐procured related queries between November 2021 and December 2024. In particular, investigation of cosmetic‐related queries was probed to capture the scale and timing of public awareness surrounding GLP‐1 RA‐associated facial changes.

## 2 Methods

Google Trends is a free tool that tracks relative search interest over time for user‐defined queries and has been used to predict behavioral changes, drug utilization, and public interest in topics like aesthetic treatments. A targeted search for the primary term “Ozempic” was conducted from November 7, 2021, to December 11, 2024, providing normalized relative search volumes (RSVs) on a 0%–100% scale. The search was conducted using “Worldwide” data with no language or regional restrictions to capture the broadest possible scope of public interest. The start date of November 2021 was strategically selected, beginning 5 months after the landmark June 2021 U.S. FDA approval of high‐dose semaglutide (Wegovy) for weight loss. Terms related to aesthetic implications from the “related queries” section were extracted and visualized. A “related query” represents a term frequently searched alongside the primary keyword, with its percentage reflecting the relative increase in interest compared to a previous period. To assess shifts in public interest in facial aesthetics linked to “Ozempic,” relevant related queries were plotted by their relative search volume increases, highlighting aesthetic‐related trends during the study period. GT data were plotted as single points and fit with a second‐order polynomial (quadratic) curve (*R* ^2^ = 0.8843). All GT data are publicly accessible and reproducible by querying the term “Ozempic” (Worldwide, all categories, Nov 7, 2021–Dec 11, 2024) and exporting weekly RSV and “Related queries” exactly as performed here.

To assess the relationship between RSV and prescribing volume, raw data was extracted from a previously published paper by Gratzl et al. using WebPlotDigitizer (Version 5.0, Automeris LLC, Austin, TX) [10]. WebPlotDigitizer is a web‐based tool that allows users to extract numerical data from images of graphs, charts, and plots by converting visual elements into quantitative values [11]. The association between variables was assessed using both Pearson's correlation coefficient (*r*) and Spearman's rank correlation coefficient (ρ) to evaluate linear and monotonic relationships, respectively.

## 3 Results

### 3.1 Google Trends Analysis

**FIGURE 1:**
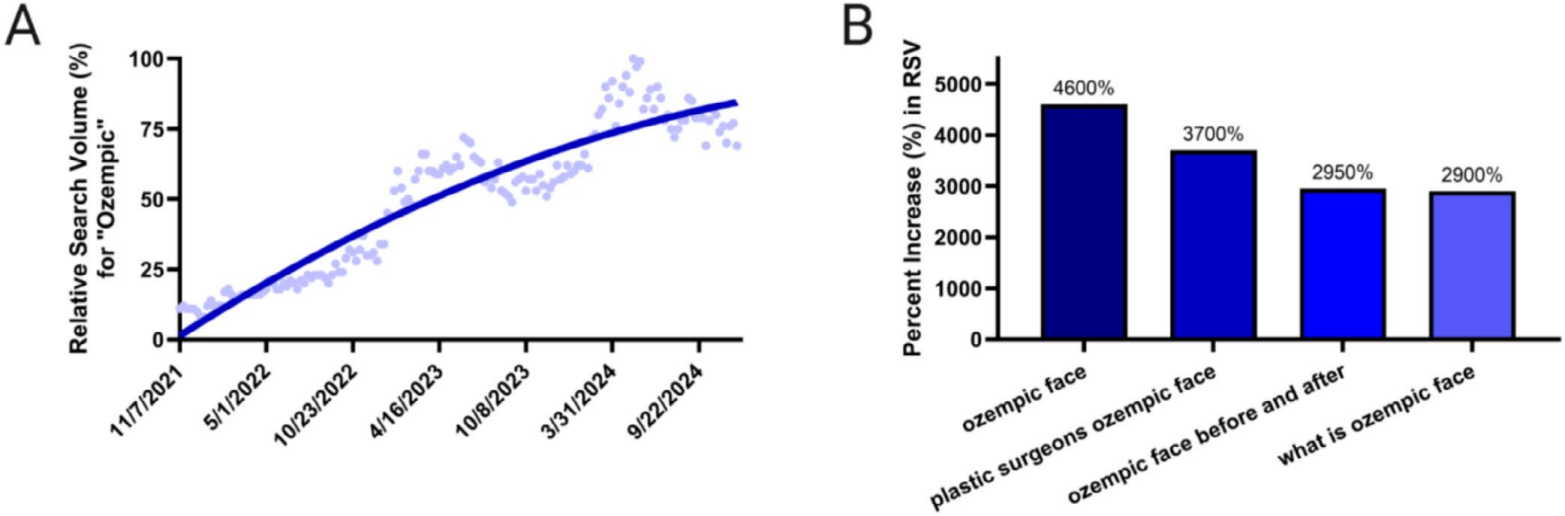
(A) Relative public search volume for “Ozempic” from 2021 through 2024 and (B) related queries relating to aesthetic side effects. Related queries are terms that are searched in conjunction with the original search term (“Ozempic” in this case). Data Source = Google Trends.

The analysis of GT data for the search term “Ozempic” from November 7, 2021, to December 11, 2024, revealed a steady increase in RSV, indicating growing public interest in the GLP‐1 RA (Figure 1A). Countries with the highest interest, which is defined as the location the search term was most popular during the specified time frame, included (in decreasing order): Canada, Australia, The United States, Ireland, Brazil, Norway, Sweden, Finland, Puerto Rico, and Belgium. Among related queries, “Ozempic face” showed the highest relative increase in RSV (+4600%). This surge, which identified “Ozempic face” as a “Rising” related query, had its primary timeline peak in early 2023, correlating directly with the steepening curve and first major RSV peak of the primary “Ozempic” search term (Figure 1A). Notably, a second, larger peak in RSV for “Ozempic face” occurred between April and March of 2024. Regional differences showed that searches for “Ozempic face” was highest in (in decreasing order): The United States, Canada, Ireland, Australia, and the United Kingdom. This was followed by “plastic surgeons Ozempic face” (+3700%), “Ozempic face before and after” (+2950%), and “what is Ozempic face” (+2900%), highlighting significant and burgeoning public attention toward aesthetic sequelae, particularly facial volume loss, associated with GLP1RA use (Figure 1B).

### 3.2 Correlation Analysis

**FIGURE 2:**
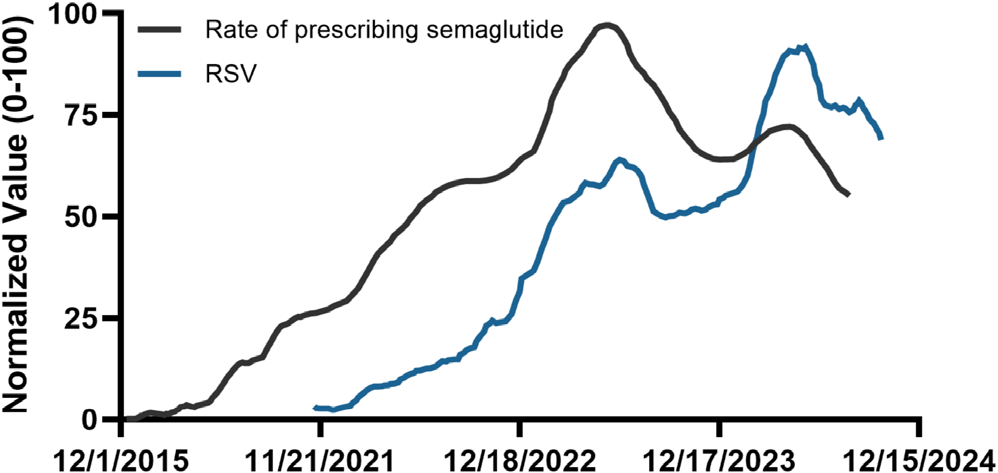
Normalized temporal trends in semaglutide prescribing volume and Google search interest. Data Source = Google Trends.

Across the monthly observations in which both datasets were present, semaglutide prescribing volume was highly correlated with RSV. Spearman's rank analysis showed a very strong monotonic association (⍴ = 0.96, *p* = 2.4 × 10^−38^), and Pearson's test confirmed an equally robust linear relationship (*r* = 0.94, *p* = 4.0 × 10^−33^). Together, these results indicate that public search interest closely mirrors adoption of the drug in clinical practice (Figure 2).

## 4 Discussion

The present infodemiologic investigation demonstrates that public interest in GLP‐1 RAs surged concurrently with an even steeper rise in searches explicitly linking the drug to facial aesthetic sequelae. The temporal coupling of trajectories, an approximately four‐fold increase in RSV for “Ozempic” accompanied by a 46‐fold increase for “Ozempic face” within the same interval, suggests that rising GLP‐1 RA searches were not driven solely by its labeled uses, but also by heightened concerns toward its cosmetic ramifications. Importantly, several of the fastest‐growing related queries (e.g., “plastic surgeons Ozempic face,” “Ozempic face before and after”) directly reference surgical or minimally invasive correction, implying that individuals who research the drug are simultaneously exploring worries about facial fat volume loss, aesthetic appearance changes with weight loss, and avenues for professional aesthetic remediation.

This search behavior aligns with contemporary consumer patterns in which patients increasingly crowd‐source health information online before seeking clinical care [12]. Our data extend this infodemiologic phenomenon to weight‐loss medication‐induced facial lipoatrophy. For aesthetic practitioners, these findings may represent a growing expectation that expertise in weight‐loss pharmacotherapy be integrated with nutritional therapies and regenerative aesthetic or volumizing solutions capable of mitigating facial soft‐tissue deficits through either direct filling or biostimulation.

### 4.1 Clinical Characterization

**FIGURE 3:**
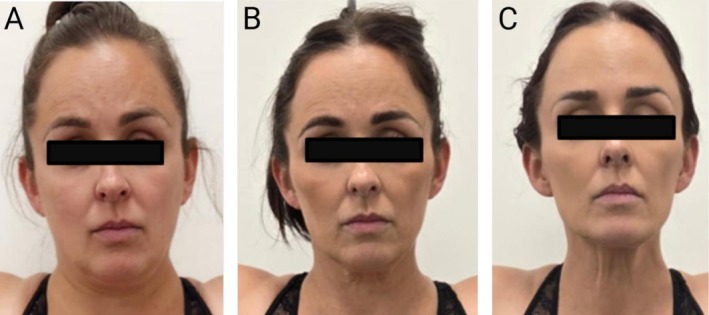
Progressive facial changes in a 42‐year‐old female patient following GLP‐1 receptor agonist (GLP‐1 RA) therapy. (A) Baseline appearance prior to treatment. (B) 11 weeks after starting GLP‐1RA after losing 23.7 lbs., showing early infraorbital hollowing, malar flattening, and mild jowling. (C) 20 weeks after starting GLP‐1RA after losing 38.6 lbs., with pronounced temporal and midfacial hollowing, visible infraorbital rim, deepened nasolabial folds, marionette lines, and cervicomental skin laxity. These findings reflect compartmental fat loss, ligament unmasking, and dermal laxity consistent with GLP‐1RA‐induced facial lipoatrophy.

Weight loss medications can trigger rapid, compartment‐specific facial volume loss that exceeds the adaptive capacity of retaining ligaments and the dermal extracellular matrix. Volume depletion is most pronounced in the deep medial cheek, sub‐orbicularis oculi fat (SOOF), and deep temporal compartments, resulting in infraorbital hollowing, temporal concavity, and A‐frame deformity, as progressively captured in Figure 3. As deep support structures collapse, superficial fat pads descend along ligaments, leading to a widened lid–cheek junction, flattened ogee curve, and formation of fixed nasolabial and marionette folds. Adipose loss in the cervicomental region sharpens the jawline but may worsen jowling. These changes collectively reflect a pattern of active facial lipoatrophy, characterized by fat pad descent, skin laxity, and accelerated signs of aging.

**FIGURE 4:**
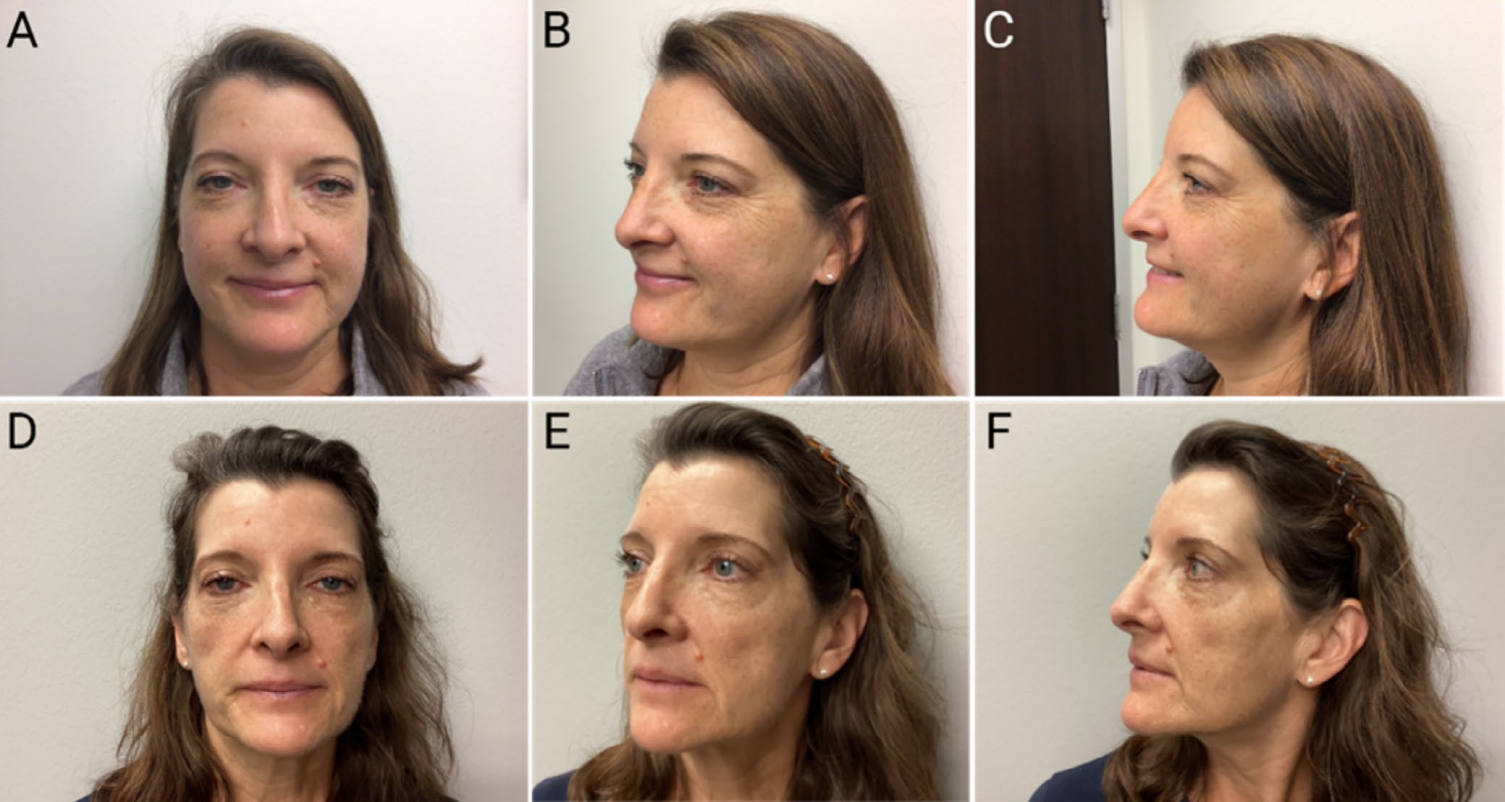
Accelerated facial aging and altered skin quality in a 57 year‐old female treated with GLP‐1 RA therapy. (A, B, C) Baseline appearance prior to treatment. (D, E, F) 28 months after starting GLP‐1 RA with significant infraorbital, submalar, and temporal lipoatrophy with moderate jowl formation. Skin quality changes include increased hyperpigmentation, uneven skin tone, and diminished skin turgor and luminosity.

In the context of significant weight loss from GLP‐1RA therapy, the volumetric changes observed from loss of facial fat compartments may also accelerate skin aging by altering skin quality. These changes may be manifested by uneven tone and hyperpigmentation as well as decreased luminosity and skin turgor, as illustrated in Figure 4. The latest clinical and mechanistic evidence indicates that these effects are driven by multiple factors, including the depletion of dermal and subcutaneous white adipose tissue, disruptions in adipose stem cell proliferation and differentiation, and alterations in the secretion and regulation of hormonal and metabolic factors [5]. Taken together, these changes compromise the structural integrity and barrier function of the skin, leading to accelerated signs of aging.

### 4.2 Therapeutic and Preventative Strategies

Among currently available interventions, regenerative biostimulators, such as CaHA or PLLA, merit particular consideration. Depending on its dilution, CaHA provides immediate volumization while also stimulating neocollagenesis, elastin production, and angiogenesis, thereby addressing both soft‐tissue volume loss and skin quality decline [13, 14]. Clinical studies have demonstrated durable restoration of malar fullness and lower‐face contour after lipoatrophy, with histologic evidence of mature type‐I collagen deposition persisting beyond the carrier gel's resorption [15, 16, 17]. CaHA's dual mechanism of direct filling and ECM regeneration may offer an immediate and long‐term answer to the catabolic effects provoked by GLP‐1RA‐mediated adipose depletion. Additional interventions that may offer immediate volumetric correction may include high elastic modulus hyaluronic acid fillers. In addition, energy‐based skin tightening devices (i.e., microfocused‐ultrasound, radiofrequency microneedling, lasers) may persist as options for targeted skin tightening.

### 4.3 A Practical Clinical Framework

Building on these principles, a practical framework can be organized around two therapeutic pathways: prophylaxis and restoration, to guide management across the continuum of weight‐loss treatment.

### 4.4 Prophylaxis Pathway

The Prophylaxis pathway is best suited for patients preparing to initiate GLP‐1RA therapy or those in the early stages of weight reduction. For individuals expected to lose ≥ 5%–10% of their baseline body weight, early initiation of biostimulatory treatment soon after starting GLP‐1RA therapy may help preserve facial volume. This recommendation is supported by recent radiographic evidence quantifying “Ozempic face,” which demonstrated an average 7% loss of midfacial volume for every 10 kg (≈22 lb) of weight lost [18].

Additionally, recent clinical data from Durairaj et al. further substantiates this approach: in GLP‐1RA users undergoing rapid weight loss, early treatment with hyperdilute CaHA‐CMC (1:3) maintained mid‐ and lower‐face volume, reduced jowling and nasolabial‐fold depth, and achieved universal patient satisfaction despite average weight losses exceeding 9% of baseline [19]. These findings reinforce the rationale for initiating collagen biostimulation once approximately 5%–10% body‐weight loss is anticipated, thereby stimulating extracellular‐matrix synthesis before overt structural deflation occurs.

Preventive CaHA‐CMC treatment can establish a collagenous scaffold that maintains facial contours throughout ongoing weight reduction. This proactive strategy combines patient counseling on anticipated changes with early regenerative intervention—targeting the superficial fat compartments, where biostimulators exert their strongest regenerative effects—to sustain soft‐tissue integrity and delay visible signs of facial lipoatrophy.

### 4.5 Restoration Pathway

For patients who present after substantial weight loss and exhibit established facial hollowing and laxity, correction should target both structural and skin quality correction.

Sarlos et al. described two post‐semaglutide cases successfully managed with PLLA followed by HA fillers for contour refinement, resulting in marked lifting, improved skin quality, and restoration of the ogee curve within 90 days [20]. This multimodal sequence exemplifies the Restoration pathway: global biostimulation for collagen regeneration and volumetric restoration with fillers. In severe cases, surgical fat grafting or facelift procedures remain appropriate adjuncts.

### 4.6 Additional Considerations

The first global, expert‐driven framework for aesthetic care of GLP‐1 RA patients recently provided critical guidelines on such patients' aesthetic care [21]. However, the convergence of pharmacologic weight‐management trends and aesthetic consequences highlights an unmet need for interdisciplinary guidelines. Nonetheless, healthcare providers prescribing GLP‐1RAs should counsel patients on potential facial volume changes and refer interested individuals to aesthetic medicine providers. Importantly, the facial volume loss associated with rapid weight loss is often difficult to reverse and should thus be maintained gradually and over time. Conversely, aesthetic healthcare providers should continue to refine and develop standardized algorithms to pre‐empt or reverse the aesthetic side effects of rapid fat loss.

## 5 Limitations

GT measures relative, not absolute, search activity and cannot distinguish between patients, clinicians, or media professionals, nor can it confirm user intent or differentiate unique from repeat searchers. Furthermore, by using a “Worldwide” search filter, regional or country‐specific temporal search behavior is not captured directly. Algorithmic sampling and normalization preclude direct comparison with prescription volumes. This analysis is also limited to Google Search and does not capture discussions on other major social media platforms that may also contribute significantly to public awareness. Future research should triangulate RSV data with prescription databases, electronic health records, and longitudinal imaging studies.

## 6 Conclusion

Public search interest in GLP‐1 RAs has escalated in lockstep with concern over its facial aesthetic sequelae, as evidenced by a 46‐fold spike in “Ozempic face” queries during the study window. These infodemiologic signals indicate that patients are increasingly interested in weight loss but worry about potential aesthetic sequelae. In addition, they show increased interest often from plastic and aesthetic medicine providers for the soft‐tissue deficits accompanying rapid, pharmacologically induced weight loss. Integrating counseling on potential facial volume changes into GLP‐1RA prescribing practices and deploying evidence‐based interventions should therefore become standard of care with an emphasis on preserving facial identity and structure. Prospective imaging and clinical studies are necessary to establish the incidence, dose‐dependency, and optimal management algorithms for GLP‐1RA‐associated facial lipoatrophy.

## Author Contributions

A.D.M.: Conceptualization, methodology, software, validation, formal analysis, investigation, data curation, writing – original draft, writing – review and editing, visualization, supervision, project administration. K.D.: Conceptualization, methodology, validation, formal analysis, investigation, writing – original draft, writing – review and editing, supervision, project administration. J.L.‐H.: Conceptualization, methodology, software, validation, formal analysis, investigation, data curation, writing – original draft, writing – review and editing. D.S.: Conceptualization, validation, formal analysis, data curation, writing – review and editing, visualization. M.D.: Validation, formal analysis, investigation, writing – original draft, writing – review and editing, visualization. A.D.: Conceptualization, validation, writing – review and editing, supervision.

## Funding

This work was supported by Merz Aesthetics.

## Ethics Statement

The authors confirm that the ethical policies of the *Journal of Cosmetic Dermatology* have been followed. This work did not involve human subjects research, as it consisted solely of an infodemiologic analysis of publicly available Google Trends data. No interventions, clinical procedures, or identifiable health information were collected. The article includes clinical photographs of two individuals, for whom written informed consent for publication was obtained prior to manuscript preparation. No additional ethical approval was required.

## Consent

Written informed consent for publication of the clinical images (Figures 3 and 4) was obtained from both patients.

## Conflicts of Interest

The authors declare no conflicts of interest.

## Data Availability

The data that support the findings of this study are available from the corresponding author upon reasonable request.
